# Creation of a Nanomodified Backfill Based on the Waste from Enrichment of Water-Soluble Ores

**DOI:** 10.3390/ma15103689

**Published:** 2022-05-21

**Authors:** Elena A. Ermolovich, Alexander L. Ivannikov, Marat M. Khayrutdinov, Cheynesh B. Kongar-Syuryun, Yulia S. Tyulyaeva

**Affiliations:** 1 Department of Applied Geology and Mining, Belgorod National Research University, Pobedy Street 85, 308015 Belgorod, Russia; ermolovich@bsu.edu.ru; 2 Department of Automated Control Systems, National University of Science and Technology “MISIS”, Leninsky Avenue 4, 119991 Moscow, Russia; cheinesh.kongar-siuriun@stud.thga.de (C.B.K.-S.); tyulyaeva@edu.misis.ru (Y.S.T.); 3 Itasca Consultants GmbH, Leithestrasse 111a, 45886 Gelsenkirchen, Germany; profmarat@gmail.com; 4 Department of Mineral Resources and Process Engineering, Technische Hochschule Georg Agricola (THGA), Herner Street 45, 44787 Bochum, Germany

**Keywords:** waste from enrichment of water-soluble ores, artificially supported mining method, backfill, activation, nanomodifier astralene, ultimate compressive strength

## Abstract

The paper analyzes losses during the development of low-value water-soluble ore deposits. The importance of development systems with backfill is shown. The use of industrial wastes of water-soluble ores to replace specially produced inert components in the preparation of backfill seems to be a good way to utilize them. The aim of the work was to create a fill mass with improved strength properties based on industrial wastes of water-soluble ores activated with a nanomodifying material. The characteristics (chemical and granulometric compositions) of an aggregate of the backfill based on the waste from enrichment of water-soluble ores are given. The validation of the hardening mixture compositions for various mining systems was carried out considering: the specified strength, the time of the artificial mass erection, the time to achieve the required strength properties of the material, which determine the possible intensity of the mining operations; method of transporting the backfill. The expediency of using a multilayer fulleroid nanomodifier astralene as a nanomodifying additive has been proved. The effect of the backfill activation with a nanomodifying additive, astralene, on the structural changes of halite wastes from the halurgic enrichment of water-soluble ores (potash) was investigated. To study the strength properties of the fill mass, the comparative analytical method was used. The strength properties of the backfill were measured in standard test periods, taking into account the intensity of hardening of the backfill material (after 7, 14, 28, 60, 90 days). To obtain reliable results, 10 backfill samples were tested at each of the scheduled dates. The shape and chemical composition of crystalline new forms were studied. Studies were performed using X-ray phase analysis and scanning electron microscopy. As a result of uniaxial compression of hardening backfill samples, the dependence of the ultimate strength on the astralene content and the hardening time were established. It has been experimentally proved that the use of a nanomodifying additive, astralene, in the backfill allows an increase in the strength properties of the created artificial mass by 1.76–2.36 times while reducing binder consumption.

## 1. Introduction

The growth of the world’s population more than twofold, from 3 billion people in 1960 to 7.7 billion people at present, has entailed a forced increase in agricultural production [1,2]. This has led to increased consumption of mineral fertilizers [3,4,5]. The growing demand for mineral fertilizers has required an increase in the production capacity of enterprises producing potash fertilizers [6]. Increasing the production of this type of fertilizer requires an increase in the extraction of potash salts at mining enterprises, which involves a larger amount of reserves in the development.

The increased consumption of potash fertilizers implies the intensification and growth of potash salt extraction, which in turn predetermines the high rates of geotechnology development in general. All of this is accompanied by industrial and environmental risks and induced disasters.

**Figure 1:**
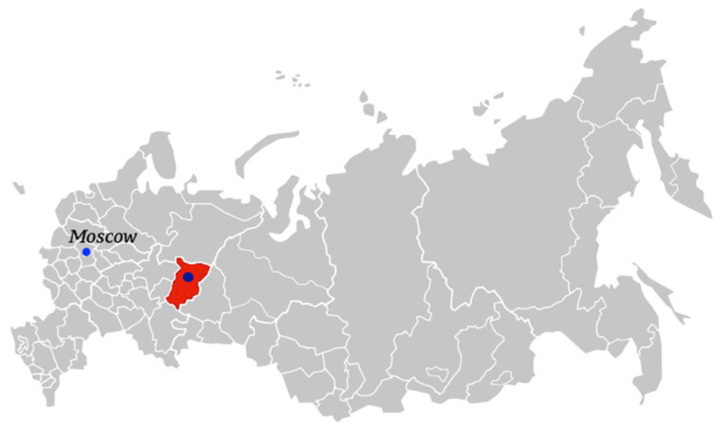
Location of the Verkhnekamsk deposit.

One of the world’s largest deposits of potassium–magnesium salts is located in the Russian Federation, in the Perm Territory (59°35′36″ N 56°48′36″ E) (Figure 1).

The Verkhnekamsk potassium–magnesium salt deposit is the main component of the Solikamsk potassium-bearing basin, located in the left-bank part of the Kama river valley. In the north, this deposit is limited by Lake Nyukhti, located in the Krasnovishersk region; in the south, it extends to the Yayva river basin. The length of the explored part of the deposit from north to south is 140 km, and from west to east, about 60 km. The thickness of the ore-bearing strata is about 80 m, and its depth is 400 m. Potash horizons are represented by alternating red layered sylvinites with rock salt interlayers. The thickness of individual potash strata ranges from 0.75 to 5 m.

**Figure 2:**
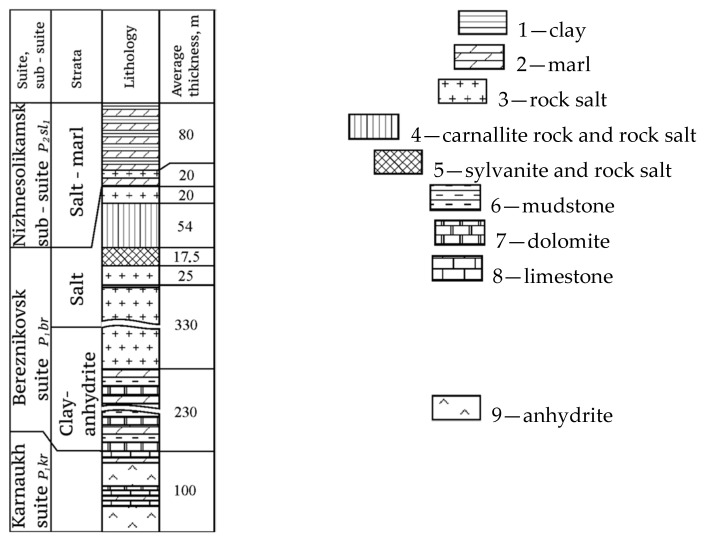
Stratigraphic section of the halogen formation of the Solikamsk depression.

The salt stratum with a total thickness of up to 550 m is subdivided (from bottom to top) into underlying rock salt (URS-P1br2), potash deposits (P1br3) consisting of sylvinite (SZ) and carnallite (CZ) zones and mantle rock salt (MRS-P1br4) (Figure 2).

All of the main reserves of the Verkhnekamsk potassium–magnesium salt deposit are located on the left bank of the Kama River. There is a small area on the right bank. The total area of the basin is more than 6.5 thousand square kilometers.

**Figure 3:**
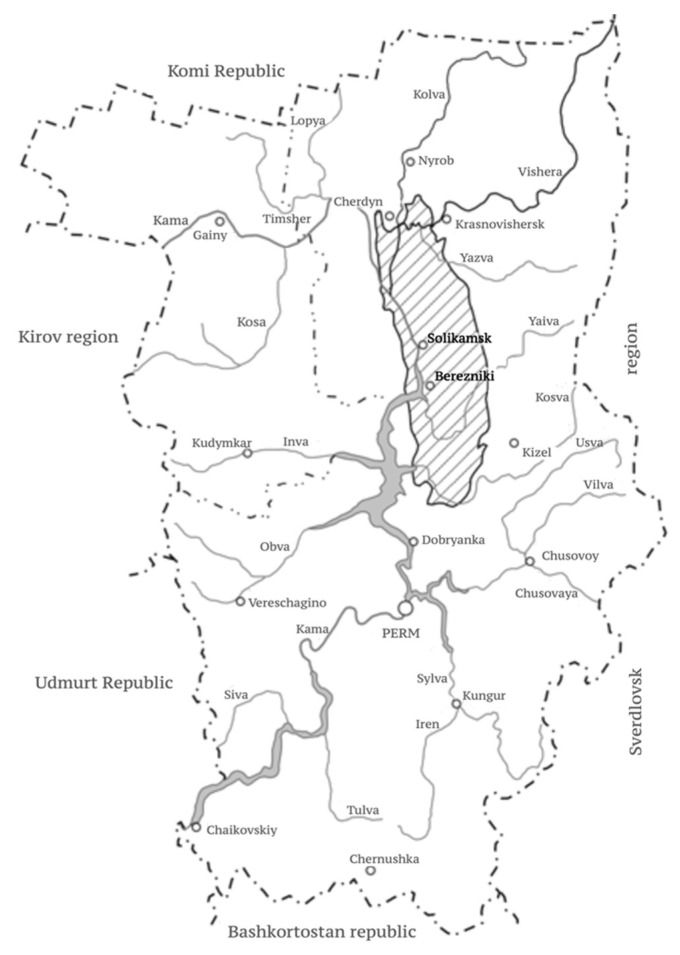
Location of the Verkhnekamsk deposit in the Perm Territory.

The Verkhnekamsk deposit was discovered in 1925, and development has been carried out by the underground method since 1934. Development centers are concentrated in the area of Solikamsk and Berezniki cities (Figure 3). At present, stope and pillar mining is used for the Verkhnekamsk deposit development.

On the basis of the above, creation of nanomodified backfill based on the tailings from enrichment of water-soluble ores, that allows replacing the traditional technology of water-soluble ore mining with a safer one and obtaining an environmental and economic effect, seems to be a very urgent task.

Stope and pillar mining are characterized by high mineral losses. This technology is most often used in the development of water-soluble ores with low value. Extraction of water-soluble ores is characterized not only by high losses (up to 65%) [7] of minerals left in pillars, but also by the formation of a large amount of waste generated during the extraction and processing of water-soluble ores. The volume of the generated waste is 60–70% of the total volume of the extracted ore mass [7].

The plasticity of natural salt pillars causes deformation changes in them, which leads to their destruction [7]. Destruction of pillars causes deformation disturbances of the overlying rock mass [8]. In some cases, the propagation of these deformation disturbances reaches the daylight surface. This leads to the formation of sinkholes and disruption of the waterproof stratum of the aquifer [9]. The violation of the waterproof stratum leads to the breakthrough of water into the mine, to its flooding and loss of reserves. Due to the destruction of rib and barrier pillars at the Verkhnekamsk deposit, deformation disturbances developed in the underworked mass and caused a breakthrough of the aquifer. As a result, two mines of the Verkhnekamsk deposit were lost. Consequently, the use of development systems that exclude or minimize the likelihood of disturbing the waterproof stratum is one of the main tasks in the development of water-soluble ore deposits.

The incessant induced impact, caused by drilling and blasting [10] and extensive exposed surfaces, causes seismic activity in the mining regions [11]. Vibrations and induced earthquakes of up to magnitude 5 are recorded at Russian and foreign mines developing deposits of water-soluble ores [12,13,14,15].

The use of development systems with artificial support reduces the likelihood of disasters and improves the qualitative and quantitative indicators of extraction. An artificial mass based on waste, while maintaining its main purpose of supporting the stoping space, allows minimizing the impact of mining enterprises on the environment [16].

Due to the limited ability of the biosphere for self-regulation and self-reproduction, it is necessary to create gentle technologies that minimize the impact of the enterprise on the environment and maintain the ecological balance [17].

## 2. Materials and Methods

Geotechnology with artificial support is impossible without the selection of backfill components that satisfy economic, technological and technical conditions [18]. The backfill is a composite material capable of hardening in mining conditions. This material contains aggregate, binder, mixing water and chemical additives.

### 2.1. Backfill

The characteristics of the future artificial mass largely depend on the properties of the starting materials. Therefore, their correct choice is one of the most important factors in the backfill technology. The material must be highly transportable, which ensures that it will be delivered through pipes over long distances without fear of premature hardening [19]. The material must have high plasticity for the most complete filling of the mined-out void. The setting time should not be less than that required to deliver the material to the stope [20]. This is especially important for materials with a large aggregate, since in this case stratification leads to an uneven distribution of the components in the mined-out void, the heterogeneity of the created artificial mass, and its reduced strength. The components of the backfill must be selected in such a way as to exclude their negative impact on the created artificial mass: loss of strength; warming up; shrinkage; expansion, etc.

### 2.2. Characteristics of Aggregate for Backfill

Due to the fact that the aggregate makes up 75–90% of the total volume of the backfill, its quality has a significant effect on the material and the artificial mass characteristics. In this regard, especially high requirements are imposed on the quality of the aggregate. In addition, large volumes of aggregate have a significant impact on the cost of the backfill, the cost of mining operations, and as a result, on the cost of the extracted ore.

Therefore, the main, widely developing direction is the replacement of the traditional, specially mined aggregate with waste from mining and processing industries. These wastes meet the following requirements: they are cheap, have stable physical and mechanical properties and a low-change granulometric composition, and are located near enterprises engaged in the extraction of minerals. With the appropriate preparation technology, these wastes will completely replace the traditional, specially mined aggregate, while maintaining the necessary characteristics of the created fill mass. Consequently, the use of waste as a replacement for traditional aggregates in the backfill composite has the potential to reduce the total cost of mining operations.

Waste from the enrichment of water-soluble ores is a product with the following properties: hygroscopicity; tendency to caking and clumping and having mainly sodium chloride in its composition. Depending on the enrichment method, the waste of water-soluble ores is divided into flotation and halurgic types. The chemical compositions of wastes differ slightly, but the difference lies in the granulometric composition. The particle size of halite waste of halurgic enrichment is 4.5 times higher.

For research and experiments, halite wastes of halurgic enrichment were used as an aggregate. Saturated salt solutions were used as a grout to avoid aggregate dissolution. The waste humidity was 10–12%. The chemical composition is given in Table 1, and granulometric composition in Table 2.

### 2.3. Binder Selection

In previous studies, various binders were used to prepare the hardening backfill: lime [21], cement [22], ash and slag waste from the State District Power Plant and Thermal Power Plant [23], blast-furnace granulated slags [24], and gypsum and calcium chloride additives [25]. In addition, in a number of studies, bischofite [26], caustic magnesite [27], magnesian cement [28], and expanded clay [29] were proposed as starting materials for the backfill material preparation. In early studies, the advantages of magnesia binders in the fill mass formation with an increased amount of salt in its composition were proved [30].

At the same time, the magnesian component of the binder increases the hardening speed and the strength of the created mass in comparison with traditional binders. Furthermore, one of the features of the magnesian binder is its ability to bind large aggregate masses with a minimum amount. In addition, magnesia binders reduce the negative effect of salt on cement. In this study, magnesia cement was used as a binder, which contained 75–85% magnesium oxide (MgO), depending on the grade.

Magnesia cement (TR (technical requirements) 5745-001-92534212-2014) is produced by mixing magnesium oxide pre-calcined to 800 °C with a 30% aqueous solution of MgCl_2_ (two weight parts of MgO per one weight part of anhydrous MgCl_2_). The main advantages of magnesia cement are fast hardening, high achievable strength, and high adhesion.

One of the largest producers of magnesia binding cements in Russia is the Russian Chromium group of companies (in the city of Beloretsk, Republic of Bashkortostan, Russia).

### 2.4. Activation of the Starting Components and Selection of the Activating Additive

Analysis of previously conducted studies of the geotechnology with backfill shows that the main cost in the backfill material is binder. Physico-chemical activation of the backfill components can improve the quality properties of the binder and, therefore, reduce its consumption.

One of the most affordable and cheap methods of activation is the mechanical method of activation in disintegrators [31,32]. In addition to mechanical treatment of the backfill material, a fairly effective activation method is the addition of activating additives to the material. Considering previous studies [33], it can be concluded that one of the most affordable and cheap activating additives can be lignosulfonate for the preparation of a backfill based on water-soluble ores. Lignosulfonate is an anionic surfactant that is a waste product of the pulp and paper industry.

Carbon frame structures (fullerenes and nanotubes) are used as additives that increase the strength of the created material. The high strength and high elasticity of nanotubes is a rather successful combination, which makes it possible to improve the mechanical properties of the material [34]. It is possible to create new nanomodified materials using the high strength characteristics and elasticity of nanotubes [35]. In this case, nanotubes act as strengthening additives. One such nanomodifying additive is astralene (TR (technical requirements) 31968474.1319.001-2000), obtained by the discharge-arc method [36,37]. Previous studies [38] have demonstrated positive results of using astralene (fulleroid multilayer synthetic nanomodifier). Its inclusion into the material significantly increases the elastic and strength properties [39]. The use of astralene as an activation additive to improve the properties of concrete-building mixtures showed positive results [40]. Water-soluble ores have hygroscopicity, caking ability, and, with a small amount of moisture, form a sufficiently dense and solid mass. Therefore, the effect of a nanomodifying additive without the use of a binder was initially studied to determine the optimal dose in the backfill.

The preparation of the material with the nanomodifying additive was carried out in the following sequence: astralene, the concentration of which is from 0.001% to 0.02% of the mass of the waste from the enrichment of water-soluble ores, is mixed for 5 min, then gaged with brine and blended for 10 min until a homogeneous mass is formed. The resulting mixtures were placed in cubes with faces of 10 cm.

### 2.5. Preparation and Study of the Backfill Material

Previously, the optimal amount of nanomodifying additive was determined, which was 0.01% of the solid mass in the material. Therefore, the amount of nanomodifying additive remained unchanged during the experimental studies. Nanomodifying additive and magnesia binder were mixed for 5 min, after which the wastes from enrichment of water-soluble ores were added, and the mixing was continued for up to 10 min. Then, the salt brine was added, and the mixing was continued for an additional 10 min until a homogeneous mass was achieved.

Such a sequence of mixing is due to a sufficiently small amount of one of the components (nanomodifying additive astralene) and will lead to its better distribution in the entire volume of the material being prepared.

Mixing was carried out in a laboratory planetary mixer MICS-D-C (МИКС-Д-Ц (EN 196-1, EN 196-3, EN 413-2, EN 459-2, EN 480-1, EN-ISO 679, NF P15-314, DIN 1164-5, UNE 80801, UNE 83258, ASTM C305, AASHTO T162). Optimal and efficient mixing was achieved due to the characteristic planetary motion of the mixer, namely, a combination of circular motion and motion around its axis. The planetary rotation speed was 62 rpm with an increase to 125 rpm at an initial circular rotation speed of 140 rpm with an increase to 250 rpm. Then, the material was placed in cubes with faces of 10 cm.

The storage and hardening of the samples occurred in conditions close to those of the mine, provided with the methodology (T = 20 ± 2 °C; W = 95 ± 5%). The subsequent compression test was carried out at specified periods in accordance with the methodology: 7, 28, and 60 days [41]. The magnitude of the ultimate compression strength of the hardened mixture was tested by crushing samples of standard sizes (edge 10 cm) on the test press PI-2000-A.

Reliability was confirmed by the repeatability of the results with a sufficient number of experiments. The condition for obtaining high reliability of the results is a large number of experiments. In order to obtain the most accurate values close to the actual ones, 18 samples were made for each composition. Then, the average values were calculated and presented in tables.

### 2.6. Microstructural Analysis of the Backfill Material

Microscopic analysis—the study of the internal structure of the created material—was carried out using optical or electronic microscopes at magnifications from 100 to 1000 or higher. The method of microscopic analysis was used to study the structure and material-mineralogical composition of the material (coarseness, various inclusions or new formations, etc., invisible to the naked eye), which made it possible to give a more detailed and accurate characterization of the material properties and quality [42].

The study of the created material microstructure required the use of analytical methods and appropriate equipment allowing adequate determination of the shape, composition and structure of particles of both the original components and new formations in the size range from tens of microns to nanometers [43,44].

To study the microstructure of the nanomodified composite prepared on the basis of wastes from enrichment of water-soluble ores, structural-mineralogical (petrographic analysis) and X-ray analyses were used.

All microstructural studies were carried out on a fracture of samples of the investigated nanomodified material. The fracture was obtained by a mechanical method. The fine delaminated fractions and dust particles, formed on a fracture as a result of mechanical influence, were removed by a jet of air.

The application of scanning electron microscopy to diagnose textured material has become the most powerful method for studying the structure and physical and chemical features of solid materials, including nanostructures, in the last few years [35,39].

Operating peculiarities and research methods using electron microscopy are analyzed in [45,46,47,48]. Scanning electron microscopes present patterns in secondary electrons, which makes it possible to highlight light and dark contours.

#### 2.6.1. Structural-Mineralogical Analysis (Petrographic Analysis) of the Backfill

Structural-mineralogical analysis (petrographic analysis) is a method of visual or microscopic investigation of the mineralogy and composition of a created material on the basis of morphological features.

Petrographic analysis was carried out on a Polam R-211 polarizing microscope using the immersion method. The phases were identified by refractive indices, birefringence, basicity, sign, elongation, and extinction angles. Immersion liquids were used as standards. The quantitative ratio of the phases (crystallographic composition) was determined by the Stroyber method. In the study using a polarizing microscope Polam R-211, the maximum magnification was 720 times.

These researches were supplemented by studying the samples using a Philips SEM 515 scanning electron microscope. In this case, the maximum magnification was 2000 at an accelerating voltage of primary electrons of 20.00 kV. The pressure in the chamber at the time of the study was 2 × 10^−5^ Torr.

#### 2.6.2. X-ray Analysis

X-ray analysis is a method of studying the structure of matter by the distribution in space and intensity of X-ray radiation scattered on the analyzed object.

A DRON-3 diffractometer was used for X-ray phase analysis. Recording signals in digital form allowed data processing automatically. Further, the obtained data were processed manually using a graphical editor or decrypted using a specially program for X-ray phase analysis of new crystalline formations. The operation of the graphical editor and the program used are described in detail in the study [49].

## 3. Results

A set of experiments were carried out to determine the optimal quantitative composition of the nanomodifying additive astralene in the backfill and its effect on the strength characteristics.

**Figure 4:**
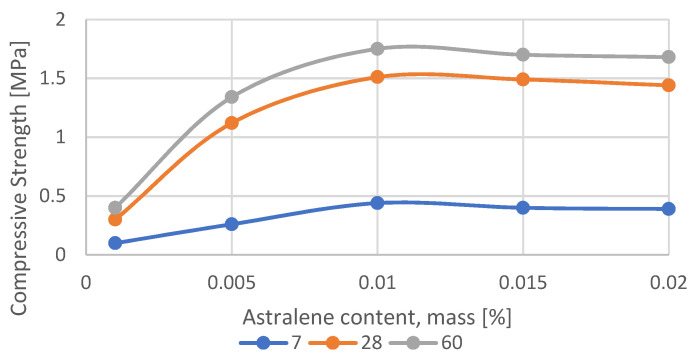
The change in the samples’ compressive strength depending on the astralene content at the age of: 7 days, 28 days60 days.

For comparison, the data obtained in reference [50] were taken when studying the effect of the activating additive astralene on the backfill based on the waste from enrichment of water-soluble ores. Experimental data on the use of the nanomodifying additive astralene are juxtaposed in Table 3 and presented in Figure 4.

### 3.1. Optimal Astralene Content

The hardened samples were tested for uniaxial compression. The test results are shown in Table 3. From the analysis of the strength characteristics of the samples, it follows that the activation with nanomodifying additive astralene significantly increases them.

The dependence of the ultimate compressive strength of the samples at the age of 7, 28 and 60 days on the astralene content *C* are very well approximated by third-order polynomial Functions (1)–(3):

σ_comp,7_ = 610,216·*C*^3^ − 27,131·*C*^2^ + 380.23·*C* + 0.0450 (R^2^ = 1.0000) (1)

σ_comp,28_ = 468,815·*C*^3^ − 21,934·*C*^2^ + 323.27·*C* − 0.0029 (R^2^ = 1.0000) (2)

σ_comp,60_ = 57,272·*C*^3^ − 3710.9·*C*^2^ + 69.226·*C* + 0.0261 (R^2^ = 0.9667) (3)

where σ_comp,7,_ σ_comp,28,_ σ_comp,60_—ultimate compressive strength of the samples at the age of 7, 28 and 60 days respectively, MPa; *C*—astralene content. mass. % of waste. Values in brackets show the accuracy of approximation R^2^, respectively.

### 3.2. Strength of Backfill with Different Component Contents

The method for selecting the composition of the hardening backfill is standard and includes studies of the main characteristics and properties. One of the main ones is obtaining necessary or specified physical and mechanical characteristics.

The choice of the rational composition of the backfill implies methods for comparing experimental compositions, analogies with previously performed works, and the exclusion of compositions that do not meet the requirements or specified characteristics.

Laboratory studies of the physical and mechanical properties of raw materials assess the possibility of their use in backfill. Then, studies of materials and hardened samples based on the selected raw materials are carried out. The samples were studied after material solidification with different component contents: binder/additive/aggregate. The components were mixed in a certain sequence in various combinations and ratios in order to determine the optimal composition of the backfill. Then, the material was fabricated into cubes with faces of 7 cm and stored in conditions close to those of the mine.

Previously, the optimal amount of nanomodifying additive was determined, which was 0.01% of the solid mass in the material. Therefore, the amount of nanomodifying additive remained unchanged during the experimental studies. The nanomodifying additive and magnesia binder were mixed for 5 min, after which the waste from enrichment of water-soluble ores was added, with mixing continued for 10 min. Then, the mixture was gaged with brine, and mixing was continued for an additional 10 min until a homogeneous mass was produced.

**Figure 5:**
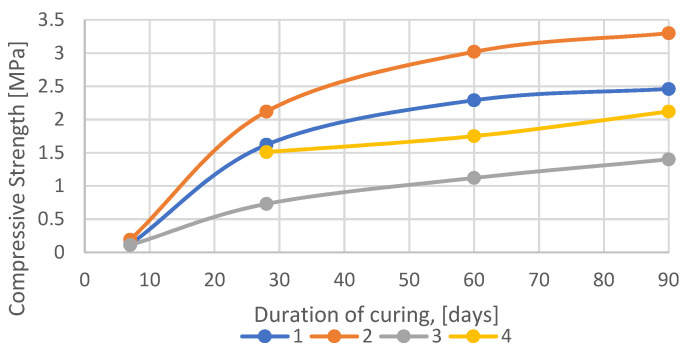
Kinetics of the backfill strength set depending on the component content: 1—waste/magnesia cement/astralene: 98.99/1.00/0.01. 2—waste/magnesia cement/astralene: 99.49/0.50/0.01. 3—waste/magnesia cement/astralene 99.99/0.00/0.01. 4—waste/magnesia cement/astralene 99.00/1.00/0.00.

The samples were tested for uniaxial compression after material hardening to determine the rational-optimal composition. The test results are presented in Table 4 and Figure 5.

The optimal water–solid ratio was selected based on the requirements that ensure the necessary mobility of the composite—20 cm according to the Suttarda viscometer.

The dependence of the ultimate compressive strength of the samples (Composition 2, Table 4) on the hardening time is well approximated by a logarithmic function:

σ_comp_ = 1.2444·ln(t) − 2.1581 (R^2^ = 0.9916) (4)

where: $\sigma_{\mathrm{comp}}$—ultimate compressive strength of the samples, MPa. t—duration of hardening, days. R^2^—accuracy of approximation.

A comparative analysis of the experimental results with the data obtained from early studies (Compositions 1a, 2a, 4a) allowed us to conclude that the use of a nanomodified additive makes it possible to reduce the magnesia binder consumption by at least 2 times while increasing the strength properties of the hardened mass. It may be also concluded that, despite some similarities to concrete, the time-dependent increase in compressive strength lasted longer than 28 days. Longer setting times resemble the case of other soil–cement composites with or without additives [51,52].

### 3.3. Microstructural Study of the Backfill Material

Structural-mineralogical and X-ray phase analyses facilitated study of the influence of a separate component of the backfill material on the creation of structural bonds. We performed X-ray phase analysis of compositions No.2, No.3 and petrographic analysis of compositions No.2, No.3 and No.4 (Table 4).

**Figure 6:**
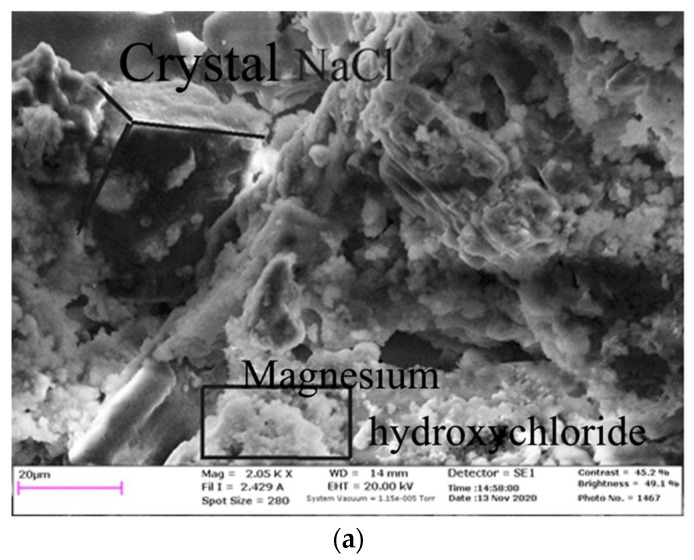

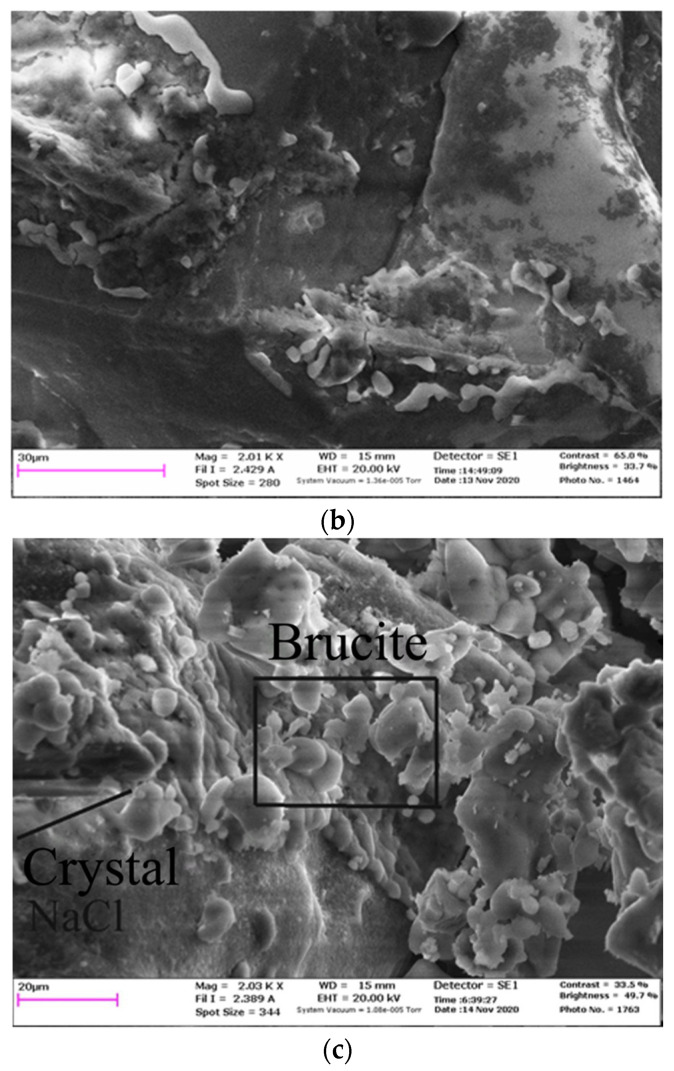
Microstructure of the studied samples. (**a**) Peripheral needle frame of magnesium hydroxy chloride. (**b**) Formation of a cryptocrystalline structural frame representing secondary NaCl crystals. (**c**) Brucite.

To determine the crystallographic parameters, we used the constants of the optical properties of minerals combined in the Winchell A.N [53,54] reference book for inorganic compounds:

- Composition No.4 (waste/nanomodifying additive): the introduction of astralene into the waste from enrichment of water-soluble ores did not cause the formation of any amorphous bonds or the appearance of new crystalline structures. This confirms the absence of any chemical interaction between these components. Astralene segregates on the surface of minerals presented in an aggregate, evenly distributing and forming continuously dense interlayers with a thickness of up to 0.5 microns in the volume of the entire composite (Figure 6a).
- Composition No.3 (waste/binder): after enrichment waste and magnesia cement mixing, amoeboid compounds are formed as a result of the concentration of the magnesian component, which is 1% of the total volume of enrichment wastes from the water-soluble ores. Mineralized crystals of waste are cemented by creating a mesh frame, the size of the faces (joints) of which varies from 10 to 15 microns. The hydrated phase of brucite (Mg (OH)_2_) is formed as a result of hydration. Brucite with a size of no more than 4–5 microns has the form of basalt plates, has a closed porosity and creates a loose structure (Figure 6b).
- Composition No.2 (waste/binder/nanomodifying additive): simultaneous introduction of a magnesian binder and a nanomodifying additive into a composite based on waste from water-soluble ores after solidification causes the formation of a continuous fine-mesh nanomodified structure. Magnesium hydroxy chlorides are structured in the form of a needle frame into the fundamental phases of the NaCl crystal matrix along the edge amoeboid formations of the Mg(OH)_2_ brucite structures. The forming crystals, the size of which along the long axis is 1–2 microns, have a needle frame. Grains of amoeboid brucite crystals have a fine-crystalline structure. The grain size does not exceed 2 microns (Figure 6c).

**Figure 7:**
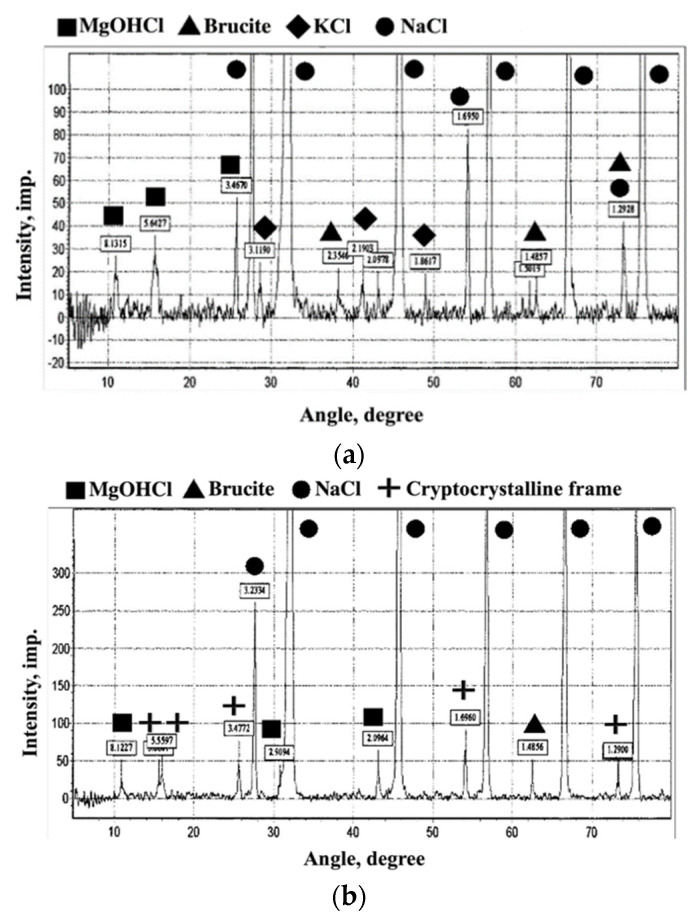
Evaluation of samples by X-ray phase method: (**a**) waste/binder, (**b**) waste/binder/astralene.

Analysis of the X-ray patterns of samples No.2 and No.3 allowed us to note that the bulk of the reflections with the highest amplitudes were crystals of sodium (NaCl) and potassium (KCl) salts, which was explained by the large amount of waste from enrichment of water-soluble ores (Figure 7a,b). Crystals of brucite and magnesium hydroxy chloride, which are the products of the magnesian binder hydration, were reflected with a lower amplitude. This proved that there is a compaction of pore voids between crystals of sodium (NaCl) and potassium (KCl) salts by filling them with brucite and magnesium hydroxy chlorides. As a result, the strength characteristics of the homogeneous mass increase during solidification.

In the studied samples, which did not contain a nanomodifying additive (Figure 7a), there were noticeably smaller numbers of magnesium hydroxy chloride reflections d_α_ = 8.1315; 5.6427; 3.4670 Å, and a slight dominance of reflections corresponding to brucite was revealed dα = 2.3540; 1.3019; 1.4857; 1.2928 Å. In samples obtained after solidification of the material containing a nanomodifying additive (Figure 7b), the numbers of reflections that corresponded to the fundamental crystal structures of magnesium hydroxy chloride were preserved d_a_ = 8.1227; 2.9094; 2.0964 Å. At the same time, there was a significant reduction in reflections corresponding to brucite d_a_ = 1.4856 Å. Additionally, in these samples, reflections appeared, indicating the formation of new structures d_a_ = 5.6577; 5.5597; 3.4772; 1.6960; 1.2900 Å, not typical for samples without a nanomodifying additive.

The use of a nanomodifying additive, astralene, influenced the formation of a fine-crystalline nanomodified structure of the fill mass. Structural-mineralogical and X-ray phase analyses made it possible to establish that astralene acts as an activating additive in the backfill. In the hardening (hydration) process, brucite was formed along the peripheral zones. This created additional stable crystal structures of magnesium hydroxy chlorides (Figure 6a) and provided an increase in the strength of the created fill mass.

In addition, the cryptocrystalline frame was formed when astralene was injected on the surface of sodium salt (NaCl) grains. The frame represented secondary crystals of these salts (Figure 6c). The formation of this structure was favored by the mutual penetration of halite aggregates and hydration products of magnesium hydroxy chlorides into the pore space and their additional adhesion.

## 4. Discussion

Figure 6a,c show the microstructures of the fill mass, visually representing the marked crystalline new formations.

In the analysis of the X-ray study (Figure 7), it can be seen that upon introduction of the nanomodifying additive astralene into the composite, reflections from the new phase appeared (Figure 7b), testifying to the new formation in the composite being created. This new formation corresponded to development of a cryptocrystalline structural frame.

**Figure 8:**
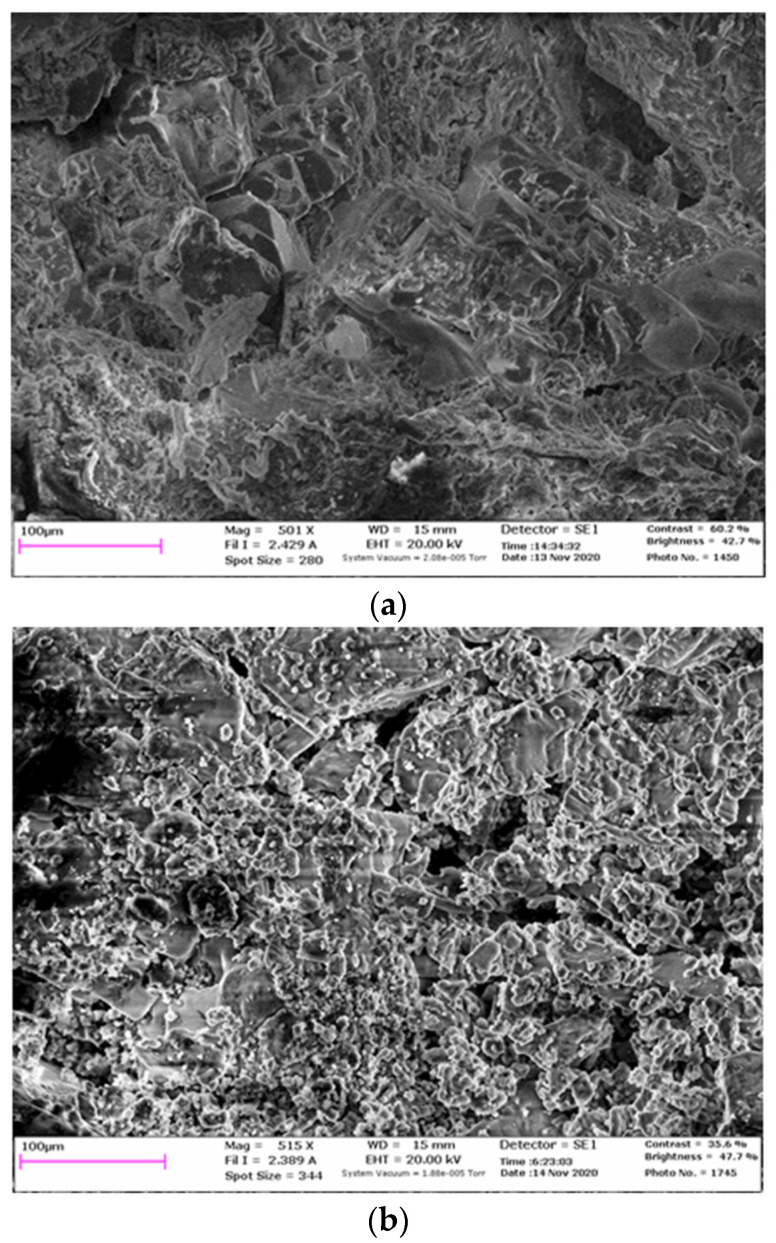
Microstructure of the studied samples: (**a**) with a modifying additive, (**b**) without a modifying additive.

Upon activation of the backfill material with astralene, after its solidification, a denser and more homogeneous structure was formed (Figure 8a), in contrast to the composite that did not contain the nanomodifying additive (Figure 8b).

When analyzing Figure 8a,b, it can be seen that image 8a is more even, while image 8b shows a sharp contrast. Dark contrasts (Figure 8b) indicate the presence of pores, and light contrasts turning into white indicate a high graininess of the material. The more even contrast in Figure 8a indicated that the composition of the material containing astralene hade less porosity and granularity. The combination of astralene with magnesian cement contributed to the formation of a dense, therefore, more durable structure. The setting time of the mixture was not changed significantly and required a long period of time. This was due to the fact that when mixing a mixture with a saturated solution of salts consisting mainly of halite, the process of hydration of magnesia binder takes a longer period of time in comparison with the setting time of magnesia-based mixtures with bischofite (saturated solution of MgCl salts).

Experiments proved that the use of the nanomodifying additive astralene in the backfill makes it possible to increase the strength properties of the created artificial mass with a decrease in binder consumption. Activation of the backfill with the additive astralene formed a fine-crystalline nanomodified structure and allowed creation of a completely new nanomodified material with stronger bonds.

Activation occurs by adding a nanomodifying additive to the backfill. The formation of a nanomodified artificial mass based on the wastes from enrichment of water-soluble ores occurs due to the formation of fine-structured bonds by filling its pore voids. As a result of the introduction of a nanomodifying additive (astralene) into the backfill, needle crystalline and cryptocrystalline frames were formed, which filled the pore space. These structures guaranteed the formation of stable structural bonds between the crystalline matrix components, which increased the strength of the mass by at least 1.76–2.36 times.

Testing of composite samples after 60 and 90 days proved that even after a standard 28 day period, an important increase in compressive strength may still be observed. The range of this increase is higher than that for standard cementitious materials such as concrete, and it is comparable to the results from the creation of soil–cement composites in the course of geotechnical works.

## 5. Conclusions

To study the possibility of creating and using nanomodified backfill material based on the waste from enrichment of water-soluble ores, the composition was selected, physical properties were studied, and micro-structural research was conducted. From the conducted research, the following conclusions can be drawn:

(1) Wastes from enrichment of water-soluble ores cannot be an ideal inert aggregate for backfill production. However, the use of magnesia cement as a binder and astralene as a nanomodifying additive will make it possible to freely use tailings of water-soluble ore enrichment for backfilling.
(2) The optimal proportion of the nanomodifying additive astralene in the backfill is 0.01% of the total mass. This content allows one to achieve maximum strength of the fill mass. The recommended waste content is 98.99% with a binder content of 1%. The use of a nanomodifying additive significantly increases the strength properties of the created backfill composite.
(3) The use of a nanomodified backfill based on waste from enrichment of water-soluble ores contributes to a multiplier effect: economic due to the introduction of mining technology that decreases losses and reduces costs for the storage of industrial waste; ecological due to reducing the volume of industrial mass and the introduction of technology that improves the safety of mining operations.

## 6. Patents

The presented results are the subject of Russian Patent RU 2754908 C1: “Backfill mixture with nanomodified additive”. Authors of the patent: Elena A. Ermolovich, Albert M. Khayrutdinov, Yulia S. Tyulyaeva, Cheynesh B. Kongar-Syuryun.

Field of application: mining industry.

Substance: Invention relates to the mining industry, namely to backfill mixtures, and can be used to backfill a goaf in the development of mineral deposits. The filling mixture contains a saturated solution of halite waste salts and a solid mixture consisting of: halite waste from potash ore processing, a binder-magnesia cement, an additive, and the filling mixture contains a nanomodified additive, astralene, as an additive. The filling mixture contains, wt.%: 11.11—a saturated solution of salts of halite waste and 88.89—a solid mixture, which contains, wt.%: halite waste from potash ore processing—98.99–99.49; nanomodified additive astralene—0.01; magnesia cement—the remainder.

Effect: increasing strength of the filling mixture, reducing the consumption of the binder in the filling mixture, increasing completeness of utilization of potash ore processing waste.

## Figures and Tables

**Table 1:** Chemical composition of waste from the enrichment of water-soluble ores (halurgic).

	Halite Waste						
Components	KCl	NaCl	MgCl_2_	CaSO_4_	Insoluble residue	Br^−^	H_2_O_cryst._
Mass fraction, %	1.91	94.3	0.07	1.914	1.7	0.026	0.08

**Table 2:** Granulometric composition of waste from the enrichment of water-soluble ores (halurgic).

	Halite Waste								Average Size
Particle size, mm	+7	7–5	5–3	3–2	2–1	1.0–0.5	0.5–0.25	−0.25	2.54
Mass fraction, %	7.4	7.3	17.0	16.3	20.9	19.5	8.6	3.0	100

**Table 3:** Compositions with different contents of astralene.

Composition	Activating Additives	Content, Mass. % of Waste	Water–Solid Ratio	Strength of Samples Under Uniaxial Compression, Mpa		
				Duration of Hardening, Days		
				7	28	60
1	astralene	0.001	0.15	0.1	0.3	0.4
2	astralene	0.005	0.15	0.26	1.12	1.34
3	astralene	0.01	0.15	0.44	1.51	1.75
4	astralene	0.015	0.15	0.4	1.49	1.7
5	astralene	0.02	0.15	0.39	1.44	1.68

**Table 4:** Research on compositions with different content of components.

Composition	Content, Mass.%				Water–Solid Ratio	Strength of Samples Under Uniaxial Compression, Mpa			
	Waste	Binder	Astralene	Ligno-Sulfonate		Duration of Hardening, Days			
						7	28	60	90
1	99.49	0.50	0.01	-	0.125	0.12	1.62	2.29	2.46
1а	98.00	1.00	-	1.00	0.125	0.10	1.20	1.70	1.80
2	98.99	1.00	0.01	-	0.125	0.19	2.12	3.02	3.30
2а	97.00	2.00	-	1.00	0.125	0.15	1.60	2.20	2.40
3	99.00	1.00	-	-	0.150	0.11	0.73	1.12	1.40
4	99.99	-	0.01	-	0.130	-	1.51	1.75	2.12
4а	99.00	-	-	1.00	0.130	-	0.80	0.90	1.00

## Data Availability

Not applicable.
